# Black soldier fly (*Hermetia illucens* L.) as a high-potential agent for bioconversion of municipal primary sewage sludge

**DOI:** 10.1007/s11356-022-20250-w

**Published:** 2022-04-27

**Authors:** Silvia Arnone, Massimiliano De Mei, Francesco Petrazzuolo, Sergio Musmeci, Lorenzo Tonelli, Andrea Salvicchi, Francesco Defilippo, Michele Curatolo, Paolo Bonilauri

**Affiliations:** 1 ENEA - Italian National Agency for New Technologies, Energy and Sustainable Economic Development - TERIN-BBC - Casaccia, Via Anguillarese 301, 00123 S. Maria Di Galeria, Rome Italy; 2 ENEA - Italian National Agency for New Technologies, Energy and Sustainable Economic Development - SSPT-BIOAG - Casaccia, Via Anguillarese 301, 00123 S. Maria Di Galeria, Rome Italy; 3 Località Canonica 44, 05018 Orvieto, TR Italy; 4 Via dei Monti di Creta 49, 00167 Rome, Italy; 5 IZSLER - Istituto Zooprofilattico Sperimentale Lombardia ed Emilia-Romagna, Via A. Bianchi, 7/9, 25124 Brescia, Italy

**Keywords:** Scavenger insects, Waste management, Municipal sewage sludge, Biorefinery, Circular economy, Green chemistry, Metals

## Abstract

The treatment of municipal wastewater produces clean water and sewage sludge (MSS), the management of which has become a serious problem in Europe. The typical destination of MSS is to spread it on land, but the presence of heavy metals and pollutants raises environmental and health concerns. Bioconversion mediated by larvae of black soldier fly (BSFL) *Hermetia illucens* (Diptera, Stratiomyidae: Hermetiinae) may be a strategy for managing MSS. The process adds value by generating larvae which contain proteins and lipids that are suitable for feed and/or for industrial or energy applications, and a residue as soil conditioner. MSS from the treatment plant of Ladispoli (Rome province) was mixed with an artificial fly diet at 50% and 75% (fresh weight basis) to feed BSFL. Larval performance, substrate reduction, and the concentrations of 12 metals in the initial and residual substrates and in larval bodies at the end of the experiments were assessed. Larval survival (> 96%) was not affected. Larval weight, larval development, larval protein and lipid content, and waste reduction increased in proportion the increase of the co-substrate (fly diet). The concentration of most of the 12 elements in the residue was reduced and, in the cases of Cu and Zn, the quantities dropped under the Italian national maximum permissible content for fertilizers. The content of metals in mature larvae did not exceed the maximum allowed concentration in raw material for feed for the European Directive. This study contributes to highlight the potential of BSF for MSS recovery and its valorization. The proportion of fly diet in the mixture influenced the process, and the one with the highest co-substrate percentage performed best. Future research using other wastes or by-products as co-substrate of MSS should be explored to determine their suitability.

## Introduction

The continuous growth of global population and urbanization is increasing the generation of waste and overexploitation of natural resources (fuels, minerals, water, land, and biodiversity). More sustainable development practices are urgently needed. Waste management is one of the main challenges of the latest decades and represents an increasingly important area of resource recovery (EEA 2020). Among wastes, urban wastewater and related sewage sludge represent a serious harm for the environment. Wastewater is a mixture of black and grey water derived from domestic activities, stormwaters, and other urban runoff. High-income countries treat about 70% of urban wastewater (UNWWDR 2017) by collecting it in sewer networks and conveying it to treatment plants with the purpose of separating clean water from the solid component, the municipal sewage sludge (MSS or biosolids). The more efficient the wastewater treatment plant and the higher the quantity of the wastewater treated, the greater the amount of MSS produced that must be managed (Bianchini et al. 2015). Currently, more than 10 million tons (dry matter) of MSS are produced per year in Europe (Eurostat 2019). The characteristics of MMS depend on the quality of the wastewater and on the treatment system. MSS is composed of organic matter (as high as 30-60%), which includes lipids (over 20%), carbohydrates (about 50%, including sugar, starch and fiber), nitrogen, phosphorus, and potassium (about 3, 1.5 and 0.7%, respectively), with a C/N ratio ranging from 10 to 20% (dry matter basis) (Kumar et al. 2017; Wei et al. 2010). This represents a potential resource for energy and valuable products (Puyol et al. 2016). However, it also can contain high levels of heavy metals (Islam et al. 2013; Kumar et al. 2017), pathogens (Clarke and Smith 2011), and physical, chemical, and biological pollutants (Strauch 1991), such as microplastics (NIVA 2018), polycyclic aromatic hydrocarbons, perfluorinated surfactants (Tavazzi et al. 2012), and polychlorinated biphenyl (Kaya et al. 2015). MSS naturally undergoes decomposition with emission of the greenhouse gases carbon dioxide, methane, and nitrous oxide (Hofman et al. 2011).

In the past, landfill and sea disposal were the most frequently used way of MSS management (EC 2001). Application of MSS on land has been considered for a long period to be the most appropriate strategy of reuse since it contributes to improvement of soil fertility and crop productivity (Sommers 1977). At present, it is the main route (50%) followed in Europe (Collivignarelli et al. 2019). On the other hand, the presence of toxic metals raises environmental and health concerns regarding long-term impact of MSS land application (Charlton et al. 2016; Elmi et al. 2020; Singh and Agrawal 2007). Evidence of persistence of heavy metals in the soil that impair microbial diversity (Chaudri et al. 1993; McGrath 1987) and affect the growth of crops and modify trophic chain (Larsen et al. 1994) formed the basis for having a precautionary approach in regulation of heavy metal concentrations in soil (Witter 1996; EC 2000). In Europe, the limit in the use of sewage sludge in agriculture is regulated by Directive 86/278/EEC. The Directives UE 850/2018 and 851/2018 introduce a new regulation for organic waste disposal, providing for the progressive reduction of the use of landfills and encouraging recycling and recovery, stating that landfilling of biosolids must be banned, land application must be limited, and other routes of recovery must be explored (Collivignarelli et al. 2019; Mateo-Sagasta et al. 2015). It has become evident that innovative technologies for MSS treatment are essential, along with attitudes and approaches that promote alternative MSS disposal and recycling options. Methods for energy production such as incineration and co-combustion (Fytili and Zabaniotou 2008), thermal treatment (Bianchini et al. 2015), pyrolysis and gasification (Manara and Zabaniotou 2012; Samolada and Zabaniotou 2014), and biological treatments (anaerobic digestion and co-digestion with other organic wastes) (Iacovidou et al. 2012; Münster and Lund 2009; Venkatesh and Elmi 2013) are being studied. Composting has also been proposed for the conversion of MSS to soil fertilizers, as is (Roig et al. 2012) or mixed with other organic waste (Dzulkurnain et al. 2017; Moretti et al. 2015; Wong et al. 2011). The process can improve the chemical and the physical properties of sewage sludge (Parr et al. 1978) but may only partially reduce the toxic heavy metal and the organic pollutant content. Furthermore, during composting, the reduction of the volume can cause an increase of some metal concentrations (Hsu and Lo 2001) and loss of biologically active nitrogen due to ammonia volatilization if it is not well ventilated (Chen 2012). Vermicomposting represents an improved bioconversion technology based on the ability of earthworms to decompose and accelerate the biodegradation process (Lim et al. 2016). He et al. (2016) observed a reduction of heavy metal concentration during vermicomposting of sewage sludge as well as did Liu et al. (2005), due to a metal sequestration carried out by earthworms. This process, known as Conversion of Organic Refuse by Saprophages (CORS) (Diener and Zurbrügg 2008), however, requires a large land area and high costs of investment (Bhat et al. 2018). Additional drawbacks are related to toxicity of MSS that can affect the survival and the activity of the earthworms (Elvira et al. 1996; Fayolle et al. 1997; Hu et al. 2021). CORS can be accomplished with another saprophagous organism that is specialized to feed on decaying organic matter, the larvae of *Hermetia illucens* (L., 1758) (Diptera, Stratiomyidae: Hermetiinae), known as the black soldier fly (BSF). *H. illucens* is native to the tropical region of South and Central America (Leclercq 1997), but has become a cosmopolitan species in tropical and warm temperate regions (Üstüner et al. 2003). The duration of its life cycle depends on nutritional and environmental conditions, but it is typically 6–7 weeks long in warm temperatures (25–30 °C) and balanced nourishment for larvae (Sheppard et al. 2002). At maturity, which normally is reached after 15–20 days, larvae gain the maximum weight, stop feeding (Bonelli et al. 2020), darken, and migrate in search of a dry area for pupation (Diclaro and Kaufman 2009; Holmes et al. 2013).

The process of biocomposting with BSF consists of feeding larvae (BSFL) with organic waste, which has the double advantage of reducing the waste to a solid residue (undigested feed, feces and exuviates) that can be used as soil amendment (Diener et al. 2011; Wang et al. 2021), and recovering added value compounds accumulated in the body of the larvae, such as proteins and lipids (Nguyen et al. 2015; Barragan-Fonseca et al. 2017; Oonincx et al. 2019). The extraction and fractioning of specific functional components (e.g., amino acids, fatty acids, minerals, and chitin) are current research topics, with a view to the development an insect-based biorefinery for production of bioplastic, bioactive peptides, chitosan, biosurfactants, coatings, or textiles (Barbi et al. 2019; Ravi et al. 2020; Neis-Beeckmann 2020). The process takes as long as the larvae need to reach the prepupal stage (15–20 days) (Diener et al. 2009; Gobbi et al. 2013). Besides a remarkable reduction of the volume of the solid waste at the end of the process (Joly and Nikiema 2019), additional benefit is the reduction of greenhouse gasses (CO_2_, CH_4_, and NO_2_) and NH_3_ emissions by over 90% compared with conventional composting (Pang et al. 2020). The voracity of the larvae and the ability of their microbiome enable them to efficiently consume a wide range of substrates (Bruno et al. 2019; Diener and Zurbrügg 2008; Jeon et al. 2011; Mazza et al. 2020; Nguyen et al. 2015). As such, there is an ongoing interest all over the world in the use of BSFL for managing organic wastes such as animal manure, and food and agroindustry waste (Joly and Nikiema 2019; Newton et al. 2005; Rehman et al. 2017a, 2017b; Singh and Kumari 2019). Kalová and Borkovcová (2013), Leong et al. (2016), Cai et al. (2018), Lalander et al. (2019), and Liu et al. (2020) used MSS as feeding substrate for BSFL, stating that there is a potentiality in bioconversion of this waste.

The wastewater treatment plant of Ladispoli, a town of 41,000 inhabitants in the province of Rome, produces about 1000 ton/year of MSS and the municipality pays more than 100 €/ton to send it to landfills for composting. Thanks to the agreement between the municipality of Ladispoli and ENEA, Flavia Acque s.r.l., the company in charge of the management of the wastewater plant of Ladispoli provided ENEA with MSS (CER code 19 08 05) for experimental trials with BSFL. A laboratory experiment was established to assess the ability of BSFL to compost MSS, using a standard fly diet, the Gainesville diet (Hogsette 1992), as co-substrate. It is widely reported that the feeding diet affects BSFL performance and impacts larval nutrient accumulation and the waste reduction (Joly and Nikiema 2019; Gold et al 2018). Moisture, pH (Cammack and Tomberlin 2017; Ma et al. 2018), macronutrient content (fats, proteins and carbohydrates), and the availability of a balanced amount of calories are important factors that influence larval development (Gobbi et al. 2013; Dortmans et al. 2017; Barragan-Fonseca et al. 2018; Barragan-Fonseca et al. 2019; Gold et al. 2018; Lalander et al. 2019). Among nutrients, protein content is considered the most important key parameter (Barragan-Fonseca et al. 2018; 2019) together with protein:carbohydrate ratios, having both a large influence on larval development and process performance (Barragan-Fonseca et al. 2020), while lipids inhibit the larval development only if provided in excess (Oonincx et al. 2015). The pH even if seems not to be a key factor for the BSFL activity (Joly and Nikiema 2019), influences BSFL activity (Meneguz et al. 2018; Ma et al. 2018) and should be in the range of 5 and 8 considered as suitable for BSFL development (Lalander et al. 2015; Rehman et al. 2017b). The optimum moisture varies between 70 and 80% (Cammack and Tomberlin 2017). Based on these evidence, we characterized the substrates for pH, for dry matter %, and macronutrient content. Furthermore, it was relevant to know the metal content of the substrates before and after the treatment. With the aim to evaluate the ability of BSFL to feed on these sewage sludge-based substrates, larval survival, final weight, and the chemical composition of mature larvae were measured. The reduction of the substrate was also detected.

## Materials and methods

### The insect

In order to have specimens for the experiments, a laboratory colony was established at the ENEA Casaccia Research Center of Rome, starting from a population of 400 BSF immature larvae provided by the Istituto Zooprofilattico Sperimentale per la Lombardia e l’Emilia Romagna of Reggio Emilia (IZSLER). We used the rearing process described by Harnden and Tomberlin (2016), which is a modified method of Sheppard et al. (2002). Larvae were fed on moistened Gainesville diet (50% wheat bran, 30% alfalfa meal, 20% corn meal; 170 ml of tap water per 100 g) (Hogsette 1992) until prepupation in a climatic chamber maintained at temperature of 25° ± 1 °C, 70 ± 10% relative humidity (RH) and darkness. Pupae were then confined in plastic boxes with lids incorporating mesh-lined holes to prevent emerging adults escaping, while maintaining 50 ± 10% RH and darkness. Newly emerged adults were housed in a cage 80 L × 80 W × 180 H cm (BugDorm,Taiwan) in a climatic chamber at 27° ± 1 °C, 70 ± 10% RH. Following Heussler et al. (2019), a photoperiod of 16L:8D cycle was set using 3 m of Inspire Ledflexi Strip Lights (Adeos Service, France) 26 W, 4000 K, 400 lumen/m. To attract fertilized females and stimulate them to oviposit preferentially in easily recoverable oviposition sites, two plastic cups were nested together with a small amount of moistened Gainesville diet inoculated with a handful of 3–4-day-old larvae in order to prevent mold growth, sandwiched between the bottoms of the two cups (Booth and Sheppard 1984; Sheppard et al. 2002). The exterior cup had a perforated bottom covered with mesh to prevent females from directly contacting the diet. Strips of corrugated cardboard were placed above the mesh allowing BSF females to lay egg masses in the small slots of the corrugated cardboard. Egg masses were incubated at 25° ± 1 °C, 60 ± 10% RH and darkness, and positioned such that the newly hatched larvae could fall on fresh Gainesville diet. Neonate larvae were maintained and reared as described above to start a new cohort. Eggs used for subsequent experiments were collected after 24-h exposure to ovipositing females, and larvae were reared for 9 days on Gainesville diet in the same rearing conditions.

### The substrates

MSS was collected directly from the wastewater treatment plant on the same day as the start of the trials. Two MSS treatments were tested: moist Gainesville diet with an equal amount of MSS mixed in (i.e., 50% MSS) and one part diet with three parts MSS (i.e., 75% MSS) on the basis of fresh weight, named S50 and S75, respectively. Moist Gainesville diet was used as control, named S0. Prior to the experiments, the total amount of the three different substrates needed for all the treatments was prepared to ensure uniformity to the mixtures.

#### Experimental design

BSFL are gregarious and voracious so it is important to balance the number of larvae with the quantity of waste to ensure maximal consumption while avoiding overcrowding and nutritional deficiency (Rivers & Dahlem 2014). For this reason, in order to set up the experimental plan, we referred to results of Tomberlin et al. (2002), Diener et al. (2009), and Parra Paz et al. (2015) who reported that larval density should be lower than 1.2–5.0 individuals per cm^2^ of surface container and estimated that the daily optimum food quantity should be at least 100 mg per larva (60% moisture content). On the basis of results of our preliminary trials (unpublished data), we choose the batch strategy, which means that we provided larvae with the total amount of feeding substrate once at the beginning of the trial, without adding or changing the substrate until the end of the process. The experimental unit consisted of 700 g of each substrate put in plastic boxes (20 L × 30 W × 9 H cm) with 500 9-day-old larvae. Larvae were separated from the stock colony rearing substrate, counted, washed, dried on filter paper and added to the substrate. In these conditions the larval density was 0.83 larvae per cm^2^ and the food availability was 1,4 g per larva. The thickness of the substrate was under 3-7 cm as recommended by Perednia (2016) and Joly and Nikiema (2019). After ensuring that the larvae had entered the substrate, the boxes were covered with perforated lids lined with a mesh size of 1 mm^2^. All boxes were placed in a climatic chamber under the same conditions for larval rearing, 3 replications each treatment (S0, S50 and S75).

### larval performance and substrate reduction

Larvae were weighed every 2–3 days after initiation (on days 2, 5, 7, and 9). Three randomized subsamples of 10 larvae each were washed, dried on filter paper, weighed, and then returned to their respective container. The trials were considered concluded when a decrease in larval weight was observed as an indication of the initiation of the prepupal stage. In fact, the maximum larval weight (Bonelli et al. 2020), as well as the observation of first prepupae (Barragan-Fonseca et al. 2018), are considered the end of the larval stage at what time mature larvae undertake the prepupal period with a non–feeding phase, accompanied by a weight loss and by the darkening of the body color (May 1961; Diener et al. 2011; Gobbi et al. 2013; Barros-Cordeiro et al. 2014; Lalander et al. 2019; Georgescu et al. 2020). So at the 4th check, all surviving individuals were separated, washed, dried, counted, and distinguished as larvae or prepupae on the basis of darkening of exoskeleton (Diclaro and Kaufman 2009; Holmes et al. 2013). Parameters and indexes were calculated as follows:

1. Larval weight (LW) (mg/larva) based on fresh weight measured at the beginning and at 2, 5, 7, and 9 days.
2. Larval survival (%), measured at the end of the trial:

$$\mathrm{LS}=\frac{\mathrm{survived}\;\mathrm{individuals}}{500}\times100$$

where 500 is the initial number of individuals for all the treatments.

(3) Prepupation (%) measured at the end of the trial counting dark larvae (prepupae):

$$\mathrm{PP}=\frac{\mathrm{prepupae}}{\mathrm{survived}\;\mathrm{individuals}}\times100$$

(4) Waste reduction (%) on wet weight basis
  $$\mathrm{WR}=\frac{\mathrm{IS}\;\left(\mathrm g\right)-\mathrm{RS}\;\left(\mathrm g\right)}{\mathrm{IS}\;(\mathrm g)}\times100$$
  where IS is the initial substrate and RS is the residual substrate.

(5) Waste reduction index (from Diener et al. 2009)
  $$\mathrm{WRI}=\frac{\mathrm{WRD}\;(\%\mathrm o\mathrm n\;\mathrm d\mathrm r\mathrm y\;\mathrm m\mathrm a\mathrm t\mathrm t\mathrm e\mathrm r\;\mathrm b\mathrm a\mathrm s\mathrm i\mathrm s)}T$$
  where WRD is waste reduction calculated on dry matter basis and *T* is the duration time of the experiment (9 days).

### Proximate analysis and pH

Samples of the substrates MSS, S0, S50, and S75 were measured for protein and lipid content at the beginning of the experiment. At the end of each trial, BSFL were frozen and then measured for protein and lipid content. Protein content was determined through sample combustion, separation of nitrogen from other gases, and then detection of nitrogen by a thermal conductivity detector (N/protein Analyzer 2000, Thermo Fisher Scientific, USA). To determine protein content, total N was multiplied with the factor Kp 6.25 for the substrate and with the factor Kp 4.76 for the insect in order to avoid the overestimation of proteins due to the presence of chitin in the exoskeleton (Janssen et al. 2017). Fat content was determined by gas chromatography based on the Caviezel method (Pendl et al. 1998). For dry matter determination (DM), all samples were incubated in an oven at 60 °C for one night. Ash content was measured by incineration at 550 °C for 4 h in a combustion oven. Carbohydrates of substrates were estimated by the difference method by subtracting from the total dry weight of the sample (100 g) the sum of moisture, lipid, protein, and the ash (g/100g_DM_):

(6) Carbohydrates (g) = 100 − [weight in g (moisture + lipid + protein + ash) in 100 g of substrate].
(7) Protein conversion ratio was calculated on dry matter basis following Lalander et al. (2019):

$$\mathrm{PrCR}=\frac{{\mathrm{Pr}}_{l}}{{\mathrm{Pr}}_{\mathrm{is}}}*\frac{{\mathrm{DM}}_{l}}{{\mathrm{DM}}_{\mathrm{is}}} \times 100$$

where Pr_*l*_ is the protein content in the mature larvae at the end of the experiment, Pr_is_ is the protein content in the initial substrate, and DM_*l*_ and DM_is_ are the dry matter (%), respectively, in the mature larvae at the end of the experiment and in the initial substrate.

At the beginning of the experiment and at each check (2, 5, 7, and 9 days after initiation), the substrates S0, S50, and S75 were measured for pH. Samples of 10 g were diluted with 17 ml of distilled water, shaken, and left until complete dissolution prior to analysis (Chan and Jang 1995) with a Metrohm E 516 Titriskop (Herisau, Switzerland).

### Metal detection

The 12 metals analyzed in this work are heavy metals, except K. As, Cd, Cr, and Pb are considered non-essential and toxic (Tchounwou et al. 2012) and pose a threat if present in large quantities. Cu, Co, Fe, Mn, Mo, Ni, and Zn are essential elements (Rengel 2004), some in traces other in abundance, and their toxicity depends on their concentration and, mostly, on their availability (Singh and Kalamdhad 2011). Measurement of metal concentration was carried out in the initial substrates, and in the residue and in larval bodies at the end of the experiment by inductively coupled plasma-mass spectrometry (ICP-MS).

According to Wang et al. (2013), the change in the concentration of metals in a substrate after composting can be expressed as the ratio between the “algebraic difference between metal concentration in the initial substrate and the final substrate” and the initial concentration × 100 on dry matter basis. Therefore, metal reduction was calculated on dry matter basis as:

7 $$\mathrm{MR}=\frac{{M}_{\mathrm{is}-}{M}_{\mathrm{rs}}}{{M}_{\mathrm{is}}} \times 100$$

where *M*_is_ and *M*_rs_ are the metal content in the initial substrate and in the residual substrate, respectively.

### Statistical analysis

Data were analyzed using PASW Statistic 17, Release Version 17.0.2 (SPSS Inc., 2008). Homogeneity of variance within groups (*n* = 3) was verified according to Levene’s test, and deviations from normality were verified by the Shapiro-Wilk’s test. When these assumptions were met, groups were compared by one-way Anova for WR and WRI of the substrates after bioconversion, BSFL protein and lipid, DM_*l*_, PrCR, and pH, and the post hoc Tukey test was applied for mean separation. In the case of slight deviations from normality and from homogeneity of variance, Welch’s *F* test and Games-Howell post hoc tests were applied. Larval weight was analyzed by the general linear model repeated measures where each box was considered as a subject of the experiment. The concentration of primary sludges was treated as a factor between the subjects, while the measurement times were the repeated factors within each subject. In the case of percentage of prepupation and MR values, the generalized linear model analysis was applied. For these data, a gamma distribution was assumed after verification by the explore procedure in the SPSS software (normality tests, overall and within groups, frequency distribution plots, comparing of probability distributions by the q-q plot). A full factorial model was used, and means and contrasts were analyzed by the Bonferroni test. Levels of significance are reported in the text and tables according to the conventional notation: ns, *P* > 0.05; *, *P* ≤ 0.05; **, *P* ≤0.01; ***, *P* ≤ 0.001.

## Results

### Larval performance and substrate reduction

**Fig. 1:**
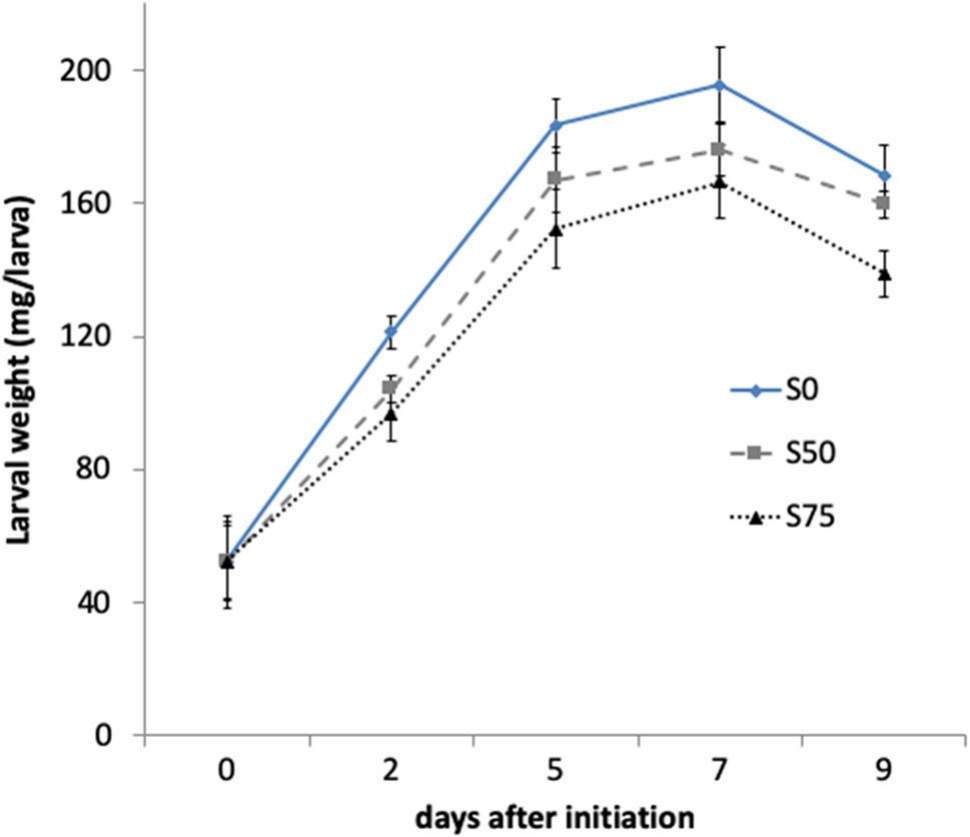
Larval weight (mg/larva) over time grown in the three substrates with different proportions of MSS (0, 50, and 75%) mixed with Gainesville diet, labeled S0, S50, and S75, respectively (bars represent standard errors)

The change in larval weight (LW) detected at each check is shown in Fig. 1. The trend of larval growth for all the treatments looks like a part of a sigmoidal curve (Sripontan et al. 2020; Laganaro et al. 2021) with a rapid weight increase from 0 to 5 days from the initiation of the experiment. The maximum LW was recorded on the 7th day from the beginning of the experiment (larvae 16-days old) equal to 176 mg/larva and 166.3 mg/larva for S50 and S75, respectively, and equal to 195.7 mg/larva for S0. A decrease was observed at the last check on 9th day from the beginning of the experiment (larvae 18-days old). The LW increase on the two substrates mixed with MSS (S50 and S75), at each check, was lower than that on the control (S0), and these differences were statistically significant according to the general linear model repeated measures analysis applied starting from the check on the 2nd day after initiation (effect between-subjects *F* = 7.303*). The substrates _*_ check interaction within-subject was not significant, suggesting that their effects were quite constant for the 3 treatments during the experiment.

At the 9th day from initiation (last check), the decrease in weight from the preceding check indicates that the larvae had entered the prepupal stage that is the post-feeding condition just before metamorphosis (Bonelli et al. 2020). In fact, for S0 and for S50, 40.6% and 48.0% of the individuals, respectively, showed a darkening of the body color (Table 1). In S75 only, the 12.0% of the individuals were changing the color, which differing significantly from values for S0 and S50 (likelihood ratio *X*^2^ 455.343*** according to the GLM). The larval survival (LS) (Table 1) at that last check was 96.8%, 98.5%, and 99.8% on S50, S75 and the control S0, respectively, and did not differ significantly.

**Table 1:** Larval survival (LS) and prepupation (PP) (%, mean ± s.e.)

	S0	S50	S75
PP^1^	40.6 ± 0.15a	48.0 ± 5.01a	12.0 ± 2.13b
LS	99.8 ± 1.00	96.8 ± 9.2	98.5 ± 7.7

^1^Values followed by different letters vary significantly at *p* < 0.05 according to Tukey’s test

As regards the % reduction of the substrate (WR% on fresh matter basis) and the reduction related to the feeding time (WRI calculated on dry matter basis), no significant differences were detected between values obtained for S0 and S50, while for the S75 treatment, both values were significantly lower than those on the other two treatments, according to Tukey’s test (Table 2).

**Table 2:** Reduction of the MSS mixed substrates after the bioconversion (mean ± s.e.)

	S0	S50	S75	F Anova
WR %	56.7 ± 0.38a	47.2 ± 3.91a	29.9 ± 1.96b	28,605***
WRI %	6.3 ± 0.04a	5.2 ± 0.75a	3.3 ± 0.46b	28,742***

Values followed by different letters in the same row vary significantly at *p* < 0.05 according to Tukey’s test

*WR* waste reduction % on fresh weight basis, *WRI* waste reduction index on dry weight basis

### Proximate analysis and pH

The proximate analysis of MSS that was used, those of the three substrates S0, S50 and S75 before bioconversion and the dry matter content in the substrates before (DM_*is*_) and after (DM_*rs*_) the bioconversion are reported in Table 3. S75 was the richest substrate both for protein and lipid content (respectively 30.3 and 4.6 g/100g_DM_) in comparison with S50 (25.1 and 4.3 g/100g_DM_) and the control diet S0 (respectively 13.9 g/100g_DM_ and 3.7 g/100g_DM_). By contrast, S75 had the lowest DM content (25.1 g/100g_DM_) compared to 36 g/100g_DM_ for S0 and the lowest carbohydrate content according to formula 6 (45.9 g/100g_DM_) in comparison with those in S0 and S50 (76.3 and 56.4, respectively).

**Table 3:** Chemical composition of the MSS that was used and of the three tested substrates before treatment and dry matter before (DM_is_) and after (DM_rs_) conversion. Values of nutrients are expressed as g/100g_DM_

	MSS	S0	S50	S75
Proteins	38.1	13.9	25.1	30.3
Lipids	5.6	3.7	4.3	4.6
Carbohydrates	30.3	76.3	56.4	45.9
DM_is_ %	22	36	29	25
DM_rs_ %		42	34	27

After the bioconversion, larval protein content (Table 4) was 36.1 ± 0.75 g/100g_DM_ and 35.4 ± 0.23 g/100g_DM_ respectively on S50 and S75, values that were significantly lower than that on S0 (39.3 ± 0.66 g/100g_DM_), while the PrCR values on S0, S50 and on S75 substrates did not significantly differ among them. As regard lipids, values showed a decrease with increasing concentration of primary sludge in the substrates even if significant differences were detected only between S75 (20.8 g/100g_DM_) and S0 (28.4 g/100g_DM_).

**Table 4:** BSFL nutrient composition (g/100g_DM_) and protein conversion rate (PrCR %) (mean ± s.e.)

		% of MSS		
	S0	S50	S75	*F* Anova
Proteins	39.3 ± 0.66a	36.1 ± 0.75b	35.4 ± 0.23b	12,585***
PrCR	38.0 ± 2.40	38.4 ± 0.33	33.0 ± 5.47	0.038 n.s
Lipids	28.4 ± 1.08a	23.6 ± 1.13ab	20.8 ± 1.72b	8,059**
DM %	30.2 ± 0.26a	29.8 ± 1.00a	26.7 ± 0.43b	8,749**

Values followed by different letters in the same row vary significantly at *p* < 0.05 according to Tukey’s test

*n.s.* not significant

**Fig. 2:**
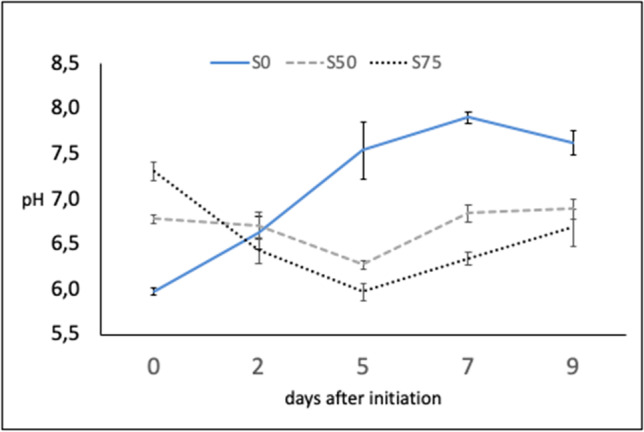
The pH variation during the bioconversion on the three feeding substrates with different proportions of MSS (0, 50, and 75%) mixed with Gainesville diet, labeled S0, S50, and S75, respectively (bars represent standard errors)). One-way ANOVA result per check (in parenthesis) among treatments: *F* (0) = 56.050, *p* = 0.001; *F* (2) 1.444 n.s.; *F* (5) = 11.902, *p* = 0.001; *F* (7) = 102.307, *p* = 0.001; *F* (9) = 12.458, *p* = 0.001

The initial pH in the mixed substrates S50 and S75 were 6.8 and 7.3, respectively (Fig. 2). These values decreased quite rapidly after infestation with BSFL, and on the 5th day, they reached the minimum (respectively 6.3 and in 6.0) before increasing until the end of the experiment. By contrast, the initial pH of the Gainesville diet S0 equal to 6.0 increased from the beginning and for three checks and then weakly decreased at the last check. Final pH approached values ranging from 6.7 for S75 and 7.6 for S0, with the latter significantly differing from S50 and S75.

### Metal reduction

**Fig. 3:**
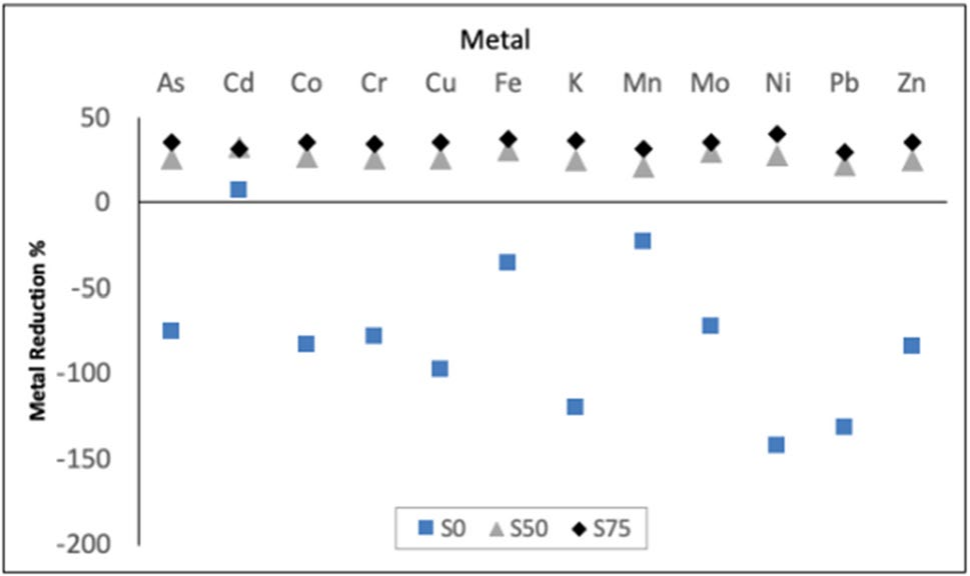
Metal reduction (%) for each metal and substrate labeled S0, S50, and S75

Table 5 shows the content of 12 metals in the three substrates before (*M*_is_) and after (*M*_rs_) the treatment with the BSFL, and in larval bodies (*M*_*l*_) at the end of the experiment. All values are expressed on dry matter basis. The highest concentration of metals was detected in the mixture (S75), with the only exception of K. The difference between the metal content in the substrates before, *M*_is_, and after BSFL treatment, *M*_rs_, according to formula 7, was expressed as metal reduction (MR) (Table 6; Fig. 3). Positive values indicate a diminution of the concentration of the metal in the residue after the BSFL treatment, while negative values indicate an increase. Values of MR for S0 were negative for all the elements except for cadmium, whereas all the values in S50 and S75 were positive without exclusion. The GLM analysis (Table 6) showed highly significant effects on MR for both factors (metal and substrate) as well as for their interaction (likelihood ratio *X*^2^ 185.557*** for substrate; *X*^2^ 32.705*** for metal; *X*^2^ 56.776*** for the interaction metal*substrate). In general, the higher the concentrations of the sewage sludge in a substrate, the higher the MR, with the magnitude of the effect varying among metals (Fig. 3) although the analysis of contrasts, according to the Bonferroni test, showed highly significant differences among the substrates only between the control S0 towards the two test substrates, S50 and S75 (*p*-value ≤ 0.001). No statistically significant differences were detected between S50 and S75 even if MR mean values were 26.47 for S50 and 34.99 for S75 (*p*-value = 0.088). All the larvae obtained from the three treatments, even those on S0, had detectable amounts of metals, *M*_*l*_, in the body (Table 5). For most of metals, *M*_*l*_ increased as the percentage of MSS in the substrate increased.

**Table 5:** Metal content (mg kg^−1^_DM_) in the initial substrates (*M*_is_), and in the residual (*M*_rs_) and in BSFL (*M*_*l*_) at the end of the bioconversion process (mean ± s.e.)

		Tested substrates		
		S0	S50	S75
As	*M*_is_	0.15 ± 0.001a	11.62 ± 0.01b	19.86 ± 0.05c
	*M*_rs_	0.27 ± 0.03a	8.62 ± 1.65b	12.75 ± 1.66b
	*M*_*l*_	0.12 ± 0.01a	0.61 ± 0.03ab	1.58 ± 0.23b
Cd	*M*_is_	0.05 ± 0.003a	0.75 ± 0.001b	1.07 ± 0.00c
	*M*_rs_	0.04 ± 0.003a	0.51 ± 0.05b	0.72 ± 0.02c
	*M*_*l*_	0.39 ± 0.06a	0.57 ± 0.33a	2.52 ± 0.33b
Co	*M*_is_	0.16 ± 0.002a	2.78 ± 0.20b	4.36 ± 0.02c
	*M*_*rs*_	0.30 ± 0.036a	1.99 ± 0.39b	2.81 ± 0.37b
	*M*_*l*_	0.03 ± 0.007a	0.06 ± 0.01a	0.15 ± 0.03b
Cr	*M*_is_	0.80 ± 0.002a	22.32 ± 0.08b	36.16 ± 0.06c
	*M*_rs_	1.43 ± 0.15a	16.48 ± 3.39b	23.56 ± 3.04b
	*M*_*l*_	0.67 ± 0.19a	0.65 ± 0.09a	1.43 ± 0.42b
Cu	*M*_is_	9.41 ± 0.02a	244.43 ± 1.21b	407.22 ± 1.33c
	*M*_rs_	18.62 ± 0.1a	181.31 ± 34.94b	262 ± 34.49b
	*M*_*l*_	19.28 ± 0.85a	21.79 ± 1.35a	37.49 ± 8.99a
Fe	*M*_is_	279.49 ± 0.81a	5341.22 ± 14.07b	8607.24 ± 29.87c
	*M*_rs_	377.35 ± 133.54a	3708.44 ± 1103.69ab	5348.25 ± 944.51b
	*M*_*l*_	131.45 ± 3.10a	195.69 ± 48.10a	435.66 ± 102.89b
K	*M*_is_	12,222.85 ± 17.03a	17,016.02 ± 118.61c	11,372.86 ± 18.38b
	*M*_rs_	26,872.28 ± 614.48b	12,774.92 ± 2472.29a	7242.17 ± 650.8a
	*M*_*l*_	9412.85 ± 347.58a	8748.19 ± 589.78a	7960.37 ± 3364.80a
Mn	*M*_is_	79.93 ± 0.59a	290.74 ± 0.40b	420.48 ± 1.33c
	*M*_rs_	97.99 ± 1.22a	228.80 ± 31.50b	288.59 ± 22.86b
	*M*_*l*_	598.49 ± 72.87a	511.21 ± 65.24a	658.65 ± 46.94a
Mo	*M*_is_	0.76 ± 0.006a	5.73 ± 0.01b	8.44 ± 0.27c
	*M*_rs_	1.31 ± 0.25a	4.06 ± 0.82ab	5.44 ± 0.77b
	*M*_*l*_	0.46 ± 0.02a	0.51 ± 0.04a	0.78 ± 0.02b
Ni	*M*_is_	1.03 ± 0.008a	16.61 ± 0.0b	28.16 ± 0.08c
	*M*_rs_	2.50 ± 0.35a	11.97 ± 2.21b	16.69 ± 2.40b
	*M*_*l*_	0.99 ± 0.27a	0.66 ± 0.03a	1.00 ± 0.38a
Pb	*M*_is_	0.53 ± 0.003a	42.13 ± 2.01b	66.19 ± 0.13c
	*M*_rs_	1.21 ± 0.16a	33.03 ± 5.91b	46.34 ± 5.20b
	*M*_*l*_	1.99 ± 0.13a	14.98 ± 2.46b	23.03 ± 3.25b
Zn	*M*_is_	60.39 ± 0.32a	783.43 ± 2.01b	1258.79 ± 5.78c
	*M*_rs_	110.98 ± 2.99a	586.22 ± 112.68b	813.19 ± 104.82b
	*M*_*l*_	161.43 ± 9.10a	169.26 ± 20.54ab	225.35 ± 10.16b

Values followed by the same letters in the same row vary significantly at *p* < 0.05 according to Tukey’s test

**Table 6:** Metal reduction (%) in the three substrates (mean ± s.e.) on dry matter basis

Metal	S0	S50	S75
As	− 74.8 ± 16.11	25.79 ± 11.52	35.78 ± 6.93
Cd	7.75 ± 1.19	32.54 ± 5.05	31.45 ± 1.78
Co	− 82.95 ± 18.75	27.12 ± 14.01	35.42 ± 7.06
Cr	− 78.30 ± 16.14	26.09 ± 12.52	34.82 ± 6.92
Cu	− 97.87 ± 1.08	25.72 ± 11.83	35.56 ± 7.06
Fe	− 35.28 ± 39.37	30.48 ± 16.94	37.79 ± 9.08
K	− 119.84 ± 3.95	24.79 ± 12.05	36.34 ± 4.62
Mn	− 22.62 ± 1.99	21.28 ± 8.89	31.36 ± 4.48
Mo	− 72.58 ± 28.06	29.25 ± 11.66	35.34 ± 7.74
Ni	− 142.06 ± 27.95	27.87 ± 10.97	40.70 ± 7.05
Pb	− 131.17 ± 26.01	21.66 ± 10.69	29.95 ± 6.52
Zn	− 83.72 ± 3.24	25.12 ± 11.82	35.33 ± 6.99

*X*^2^_metal_ = 32.705***; *X*^2^_substrate_ 185.557***; *X*^2^_interaction_ 56.776***

### Comparison to other studies

In order to verify if our results could be considered satisfactory and if there is room for improvement, some larval life history traits and valorization variables are compared with those obtained in other experiments, similar to the ours, with BSFL fed with sewage sludge or other different feed resources (Table 8).

## DISCUSSION

Results indicated that the presence of MSS in the diet influenced larval performance. In fact, larval growth and prepupal development time had an inverse relationship with the amount of MSS in the diet. This agrees with the results of Laganaro et al. (2021) with degassed sludge that increased the cost for maintenance as the sludge content increased in the diet. Larvae showed a similar growth trend on the three substrates: weight increase at the beginning of the experiment and diminution of larval weight at the 4th check (Fig. 1). So, the end of larval stage corresponded to 16–17 days (between 3rd and 4th checks) on all the substrates, that is in the range of 12 and 21 days reported on various substrates (Table 8). The maximum weight per larva was 176.0 and 166.30 mg on S50 and S75, respectively, values that, even if lower in comparison with the control (195.7 mg/larva) or with other results (Table 8), demonstrated that MSS-based substrates sustained larval growth. The highest percentage of prepupation was achieved on S50 (48.0%). The negative influence of S75 on larval development with 12% of prepupae and the lowest average weight on this mixture lead to consider, in the conditions of this work, the S50 as the more suitable co-substrate mixture for BSFL development. Instead, as it was expected from results of preliminary test (unpublished data) and according to other authors (Table 8), larval survival was not affected by the presence of MSS in the diet with values that overcome 96% on all the substrates.

The pH of MSS that was used was 7.62. The pH of the Gainesville diet used as co-substrate was 6.0 and acidified the pH of the two sludge-based substrates that resulted to be equal to 6.8 (S50) and 7.3 (S75). These values were in the recommended range for BSFL development that is between 5 and 8 (Lalander et al. 2015; Ma et al. 2017; Rehman et al. 2017b). During the process, a different variation of the trend of the pH between the MSS-based substrates and the control S0 was observed (Fig. 2) with a similar tendency for the two MSS-based mixtures (Fig. 2) to an initial decrease of the pH followed by a slow increment until the last check. On the contrary, on the control diet S0, pH underwent to a continuous increase from the beginning of the experiment until the end of the larval stage (3rd check). We did not carry out the control with no larvae, in our conditions impossible because of mold pollution; however, the trends observed suggest a probable mutual influence between larval activity and the pH of the substrates with a final adjustment to values close to neutral (6.9 on S50) or slightly acid (6.7 on S75) or slightly basic (7.6 on S0) that mirrored the results of Ma et al. (2018).

According to Barragan-Fonseca et al. (2020), high larval performance can be achieved on substrates with a protein concentration between 10 and 15% (dry matter basis) and with a carbohydrate concentration between 10 and 60% (dry matter basis) and a protein:carbohydrate ratio in the range between 1:2 and 1:4. In this respect, our mixtures had a high protein content, over the range. In fact, the MSS-based mixtures had a protein content equal to 25.1 g/100g_DM_ and 30.3 g/100g_DM_ on S50 and S75, respectively. With the carbohydrate content equal to 56.4 g/100g_DM_ (S50) and 45.9 g/100g_DM_ (S75) (Table 3), the protein:carbohydrate ratios was equal to 1:2 and 1:1.5, respectively, slightly unbalanced as regard S75. In accordance to the fact that lipids are a typically minor compound of biowaste (Gold et al. 2018), the concentration in S50 and in S75 was very low (respectively 4.3 and 4.6 g/100g_DM_). In these conditions, larvae valorized the two sludge-based substrates keeping a high protein conversion rate (Table 4). This means that they were able to utilize proteins in the same way as on the control, achieving values of accumulated proteins in S50 and S75 equal to 36.1 and 35.4 g/100g_DM_, respectively, that are higher than that obtained on primary sludge by Lalander et al. (2019) and comparable to those observed on other waste products (Table 8). Larvae were also able to accumulate lipids with no difference between the 23.6 g/100g_DM_ for S50 and 20.8 g/100g_DM_ on S75. These values were higher than the very low content obtained on the low nutrient spent coffee grounds (7 g/100g_DM_) (Permana and Ramadhani Eka Putra 2018) and on mixed sewage sludge (8.6–17.0 g/100gDM) (Raksasat et al. 2021), but lower than that on livestock waste (35 g/100g_DM_) (Sheppard et al. 1994) (Table 8).

**Fig. 4:**
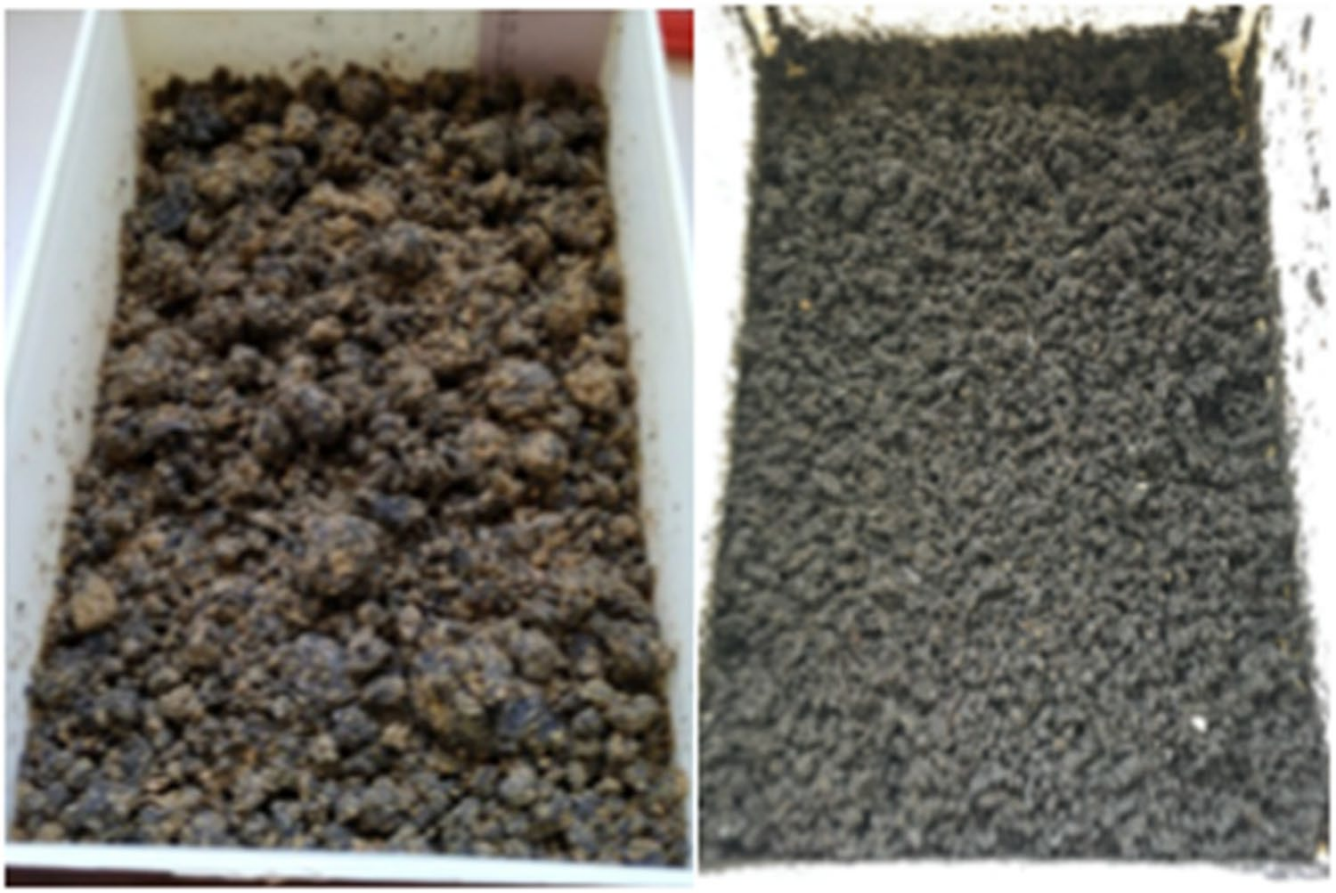
Feeding substrate S50: before (left) and after 9 days (right) of bioconversion with *H. illucens*

For both S50 and S75, the dry matter in the initial substrates, 29 and 25%, respectively, were in the range suggested as proper for a good waste reduction (moisture 70–80%) (Cammack and Tomberlin 2017). Waste reduction and the loss of humidity of the waste represent two of the main objectives of the waste bioconversion and the values obtained in this work could encourage its application for the management of MSS. In particular, we obtained, a waste reduction of 47.2% (fresh weight basis) on S50 with an increase of the dry matter from 29 to 34% suggesting that the physic-chemical properties of the S50 substrate allowed a good bioconversion. Additionally, we detected a diminution of the content of all 12 metals in the two sludge-based substrates S50 and S75 after the bioconversion with BSFL (Tables 5 and 6) without significant differences but, with a slight superiority in favor of S75, visible in Fig. 3. Furthermore, in both the initial substrates S50 and S75, Cu and Zn content exceeded the maximum limits set by the Italian law regarding the revision of the fertilizer framework (Table 7). In the residual substrates, the amount of these metals dropped drastically, which could permits high levels to be lowered below maximum allowed concentrations in fertilizers according to the Italian D.Lgs. 29 (Table 7). For what concerns the larval metal content, we detected the greatest amount of metals, with exclusion of K, in the body of larvae fed on S75 and the lowest in that of larvae fed on the control diet (Table 5). The values detected in larvae growth on S50 were for most elements in the middle. An active uptake regulation in larvae of *H. illucens* has been reported for As, Cd, Pb, and Zn (Diener et al. 2015; Bulak et al. 2018; Biancarosa et al. 2018). However, in our study, for As, Cd, and Pb, as well as for Cr, Cu, and Ni, the larval content did not exceed the limits in raw materials for feed of the European Community Directive 2002/32 (EC 2002) or of the National Research Council (NRC 2005) (Tables 5 and 7) and larvae can conversely represent a source of Co, Cr, Fe, K, Mn, Mo, and Zn that are nutritionally essential elements for living organisms, often added to diets (Coomer 2014; EFSA 2009, 2014, 2016, 2019; FDA 2021). Furthermore, the heavy metal concentration in extracted oil from larval bodies should be less than 1% of the total (Cai et al. 2018) that represents a good chance for a safe oil extraction from larvae fed on sewage sludge-based substrates (Table 8). To end, BSFL digestion changed the rough and sandy-loam texture typical of the primary sewage sludge to a fine textured matrix (Fig. 4).

**Table 7:** Values of maximum metal concentration (mg kg^−1^_DM_) allowed in fertilizers and in raw material for feed

Metal	Limits in fertilizer^1^	Limits in raw materials for feed^2^
As	-	2–30^ab^
Cd	1.5	2–15^a^
Cr	0.5	0–15^a^
Cu	230	40^b^
Ni	100	4.05^b^
Pb	140	5–400^a^
Zn	500	-

^1^Maximum allowed concentrations in fertilizers according to the Italian D.Lgs. 29 aprile 2010 n. 75

^2^Maximum allowed concentrations in raw material for feed:^a^EC 2002; ^b^NRC 2005

**Table 8:** Comparison among some bioconversion process variables obtained in this study on S50 and S75 substrates (2 first lines; mean ± se) and best results or ranges obtained by others authors studying BSFL growth on different substrates

	Mature larvae (day)	LW (mg/larva)	LS (%)	WR (%)	PrCR (%)	BSFL protein (g/100_DM_)	BSFL lipids (g/100_DM_)
S50	16–17	176.0 ± 7.81	96.8 ± 9.2	47.2 ± 3.91	38.4 ± 0.33	36.1 ± 0.75	23.6 ± 1.13
S75	> 17	166.3 ± 10.09	98.5 ± 7.7	29.9 ± 1.96	39.3 ± 5.47	35.4 ± 0.23	20.8 ± 1.72
	16–21^a1^	137^a1^	81.0 ^a1^	63.3^a1^	15^a1^	16.9^a1^	7.6^b^
	18^a2^	252^a2^	89.3^a2^	60.5^a2^	46.3^a2^	33.9^a2^	
	16.6^c^	121.3^c^	71–99^d^	41.8^c^	17.5–45.8^e^	38.1^c^	35^d^
	12–19^f^	90.8–219.8^f^	90.0–99.7^ g^	58.4–64.1^ g^		42^d^	8.6–17.0^ h^
		46.9^ h^	46.6^i^	67.4–84.8^ h^		39.9–43.1^f^	
						38–46^ l^	

*LW* larval weight, *LS* larval survival, *WR* waste reduction, *PrCR* protein conversion rate

Reference: Substrate

^a^Lalander et al. (2018): ^1^Primary sludge; ^2^dog food

^b^Permana and Ramadhani Eka Putra (2018): Spent coffee ground

^c^Diener et al. (2009): Chicken feed

^d^Sheppard et al. (1994): Livestock waste

^e^Sideris et al. (2021): Beverage by-products

^f^Spranghers et al. (2017): Different substrates

^g^Cai et al. (2018): Mixed sewage sludge

^h^Raksasat et al. (2021): Mixed sewage sludge

^i^Liu et al. (2020): Sewage sludge as is

^l^Oonincx et al. (2015): Food manufacturing by-products

## CONCLUSION

This study investigated the possibility of applying the bioconversion technology mediated by BSFL to support the management of municipal sewage sludge produced by the treatment plant of Ladispoli, a town in the province of Rome. The use of a co-substrate influenced the suitability of MSS for feeding BSFL, since lower is the MSS in the larval diet higher is the performance of the bioconversion. In fact, the mixture at 50% gave best results in terms of larval biomass, larval protein, and lipid concentration and for the major reduction of the substrate. This suggests however that the blend of municipal primary sludge with other organic substrates, such as organic urban waste and agri-food waste, for a bioconversion with BSFL could be a helpful application. At the end of the process, larvae had a valuable concentration of proteins and lipids, that means they could convert the feeding substrate in a raw material which can be inserted in a biorefinery process for the production of a wide range of compounds for industry (biosurfactants, bioplastic, detergents, cosmetics, bioactive molecules such as antimicrobial peptides, medium chain fatty acids) and for energy purpose (biofuel). The lowering of the concentration of heavy metals in the residue of the two mixtures indicates that this strategy can also be considered useful for a safe bioconversion of biosolids containing heavy metals and for the use of the residue as amendments or fertilizers. In this case, the reduction of the metal content was greater the higher was the concentration of sludge in the initial substrate (75%), suggesting that may make sense varying the percentages depending on the purpose of the application, monitoring concurrently larval accumulation for heavy metals and for those elements that, as nutritionally essential, would be useful to recover. The comparison of the results with the control diet and with data of other works leads to believe that there should be room for improving larval weight, their lipid content, and the waste reduction efficiency, considering the use of different co-substrates with a balanced supply of nutrients. With these aims, future research could take steps to evaluate different percentages of mixtures of MSS with different kind of bio-waste as well as different starting with larvae of minor age and/or a lower or higher number of larvae per unit weight of substrate. Since there should be a mutual influence between BSFL activity and pH of the substrates, the initial pH of the two components, biosolids, and the wastes added as co-substrates, and of the prepared mixtures for bioconversion must be taken into account in order to monitor that pH does not deviate too far from neutral values. This impacts on the final pH of the residue that will be used as soil amendment or fertilizers. MSS had a high protein and water content. This suggests that it would be favorable to mix the MSS with bio-waste reach in carbohydrates as fruit and vegetable waste from agri-food market and industry. Since biosolids represent an important fraction of municipal waste, the use of mixtures of biosolids with other organic wastes should be encouraged in a scenario of circular economy approach and integrated waste management. This could represent a potentially valuable solution for municipality that could involve local productive realities in a view of a participatory territorial approach.

## Data Availability

The datasets generated and/or analyzed during the current study are available from the corresponding author on reasonable request.

## Abbreviations

BSFL: Black solder fly larvae
DM: Dry matter (%)
DM_is_: Dry matter of initial substrate (%)
DM_rs_: Dry matter of residual substrate (%)
DM_*l*_: Dry matter of larvae (%)
GLM: Generalized linear model statistic test
IS: Initial substrate (g)
LS: Larval survival (%)
LW: Larval weight (mg)
*M*_is_: Metal content in the initial substrate (mg kg^−1^_DM_)
*M*_*l*_: Metal content in larvae after bioconversion (mg kg^−1^_DM_)
*M*_rs_: Metal content in the residual substrate (mg kg^−1^_DM_)
MR: Metal reduction (%)
MSS: Municipal sewage sludge
PrCR: Protein conversion ratio (%)
RS: Residual substrate (g)
WR: Waste reduction (% fresh weight basis)
WRI: Waste reduction Index
